# Homogeneous monocytes and macrophages from human embryonic stem cells following coculture-free differentiation in M-CSF and IL-3

**DOI:** 10.1016/j.exphem.2008.04.009

**Published:** 2008-09

**Authors:** Karl R. Karlsson, Sally Cowley, Fernando O. Martinez, Michael Shaw, Stephen L. Minger, William James

**Affiliations:** a Sir William Dunn School of Pathology, University of Oxford, South Parks Road, Oxford, UK; b Stem Cell Biology Laboratory, Wolfson Centre for Age Related Disease, King's College London, London UK

## Abstract

**Objective:**

To develop a simple and efficient method for producing homogeneous populations of monocytes and macrophages from human embryonic stem cells (hES).

**Materials and Methods:**

Human embryonic stem cell lines KCL001, KCL002, and HUES-2 were differentiated into monocytes by coculture-free differentiation with two growth factors using a three-step method. The method involved embryoid body (EB) formation in hES media, directed differentiation with macrophage colony-stimulating factor and interleukin (IL)-3, and harvest of nonadherent monocytes from the culture supernatants. hES monocytes (esMCs) were analyzed by microscopy, flow cytometry, transcriptome analysis, and tested for the ability to differentiate into macrophages. hES monocyte–derived macrophages (esMDM) were analyzed for phagocytosis and endocytosis by microscopy and flow cytometry, cytokine secretion by multiplex cytokine assay, and for interferon (IFN)-γ and IL-4 activation by flow cytometry.

**Results:**

Homogeneous esMCs (>90% CD14-positive) that did not require any additional purification steps were produced after 18.7 ± 7.7 days (mean ± SD, n = 19). Production continued for several months when growth factors were replaced, with a total yield of 3.4 × 10^5^ ± 2.0 esMCs (mean ± SD, n = 9) per EB. Transcriptome analysis of the esMC and the esMDM revealed a distinct myeloid signature that correlated with primary adult blood–derived monocytes and spleen tissue samples but not with other tissue samples tested. We found that esMCs and esMDMs expressed well-defined markers of the mononuclear phagocyte system including PU-1, C/EBPα, EMR1, and EMR2, MPEG1, CD1c, CD4, CD18, CD32, CD33, CD68, cathepsins and serine carboxypeptidase. Finally, esMCs differentiated into functional macrophages that could endocytose acetylated low-density lipoprotein, phagocytose opsonized yeast particles, secrete specific cytokines in response to lipopolysaccharide, and be activated differentially with IFN-γ and IL-4.

**Conclusions:**

We have developed a simple and efficient method for producing homogeneous populations of monocytes and macrophages from hES cells. esMCs have a myeloid signature and can differentiate into functional macrophages. The method should prove useful in answering experimental questions regarding monocyte and macrophage development and biology.

The mononuclear phagocyte system consists of cells derived from progenitor cells in the bone marrow. These myeloid progenitor cells differentiate into monocytes, which enter the circulation and migrate into various tissues where they differentiate into macrophages [1]. Mononuclear phagocytes as they appear in tissues share certain features, including morphology, expression of enzymes and receptors, endocytic and phagocytic capability, and secretion of cytokines in response to pathogen stimuli [2]. However, mononuclear phagocytes are phenotypically and functionally heterogeneous as a result of cellular differentiation, tissue distribution, and activation by exogenous stimuli such as the T-cell cytokines interferon (IFN)-γ and interleukin (IL)-4 [3].

Monocytes and macrophages play key roles in various diseases including atherosclerosis, sepsis, cancer, tuberculosis, and HIV-1 [4]. Although advances in gene knockout technology in mouse have led to major contributions to the knowledge of murine macrophage development and biology [5,6], the human system differs extensively from mouse and remains poorly understood due to limitations in current technologies.

Several methods have been developed for generating human primary monocytes and macrophages for in vitro studies, but methods differ in cell yield, purity, and activation status of cells, resulting in contradictory findings [7]. Furthermore, the tendency of monocytes and macrophages to remain in a G_0_-state and to degrade exogenous macromolecules makes them inherently difficult to transfect. Together, this limits the ability to genetically manipulate human monocytes and macrophages and to investigate their development and biology.

Pluripotent embryonic stem (ES) cells offer an attractive alternative to overcome these problems. Previous work has indeed shown that mononuclear phagocytes, including macrophages and dendritic cells, can be generated from both mouse [8–12] and human ES (hES) cells [13,14]. However, current methods using hES cells are complicated by the requirements for cocultures with cell lines, use of complex cytokine cocktails and additional purification steps, which limits their use in studies requiring high numbers of homogenous cells.

Here we describe a simple and efficient method for producing homogeneous monocytes from hES cells that does not require additional purification. Following differentiation with macrophage colony-stimulating factor (M-CSF) and IL-3, a homogeneous population of CD14-positive monocytes is produced. These hES monocytes (esMCs) have a distinct myeloid signature and are capable of differentiating into functional macrophages. The method should prove useful in answering experimental questions regarding human monocyte and macrophage development and biology especially when combined with specific genetic modification of hES cells.

## Materials and methods

### Culture of hES cells

The human ES cell line HUES-2 (passages 16–38) was obtained from the HUES Facility, University of Harvard [15]. The hES cell lines KCL001 (passages 5–25) [16] and KCL002 (passages 12–40) were derived at the Stem Cell Biology Laboratory, King's College London. Work on all three cell lines was reviewed and approved by the UK Stem Cell Bank Steering Committee. The generation of hES-derived monocytes and macrophages was confirmed using all three cell lines. Data presented here were from HUES-2 ES cell line unless otherwise indicated in the figure legend. HUES-2 ES cells were cultured at 37°C, 5% CO_2_ and atmospheric O_2_, and maintained on mitomycin C-inactivated mouse embryo fibroblasts (MEF) in 0.1% gelatin-coated six-well tissue culture plates containing 2 mL hES medium. hES medium consisted of knockout Dulbecco's modified Eagle's medium (Invitrogen GIBCO), 10% KO-Serum Replacement (Invitrogen GIBCO), 0.5% albumin (cat. no. 534021-4l; Sigma, St Louis, MO, USA), 2 mM Glutamax-I (Invitrogen GIBCO), 1% nonessential amino acids (Invitrogen GIBCO), 100 U/mL penicillin and 100 μg/mL streptomycin (Invitrogen GIBCO), 0.055 mM β-mercaptoethanol, 10 ng/mL basic fibroblast growth factor (R&D). Medium was changed daily by removing 1 mL and replacing with fresh hES medium. hES colonies were typically passaged weekly after assessment of size and morphology. Undifferentiated colonies were microdissected using a heat-pulled glass pipette and colonies transferred to MEFs that had been conditioned with hES medium overnight.

### Differentiation of hES cells into monocytes

**Figure 1:**
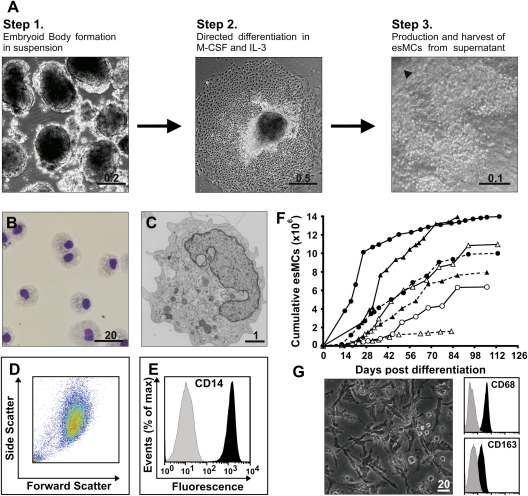
A three-step method for the differentiation of human embryonic stem (hES) cells into monocytes. (**A**) Human hES cells were removed from mouse embryonic fibroblasts either by microdissecting colonies or treating with trypsin. Patches were cultured with hES medium in suspension for spontaneous embryoid body (EB) formation for 3 days (Step 1). Adherent EBs were next differentiated in macrophage colony-stimulating factor (M-CSF) and interleukin (IL)-3 (Step 2). hES monocytes (esMCs) were produced and harvested from the supernatant of cultures around day 18, and growth factors replaced for continuous esMC production (Step 3). Arrow head in Step 3 show part of an EB. Images are from hES cell line KLC002 and representative of KCL001 and HUES-2. (**B**) Eosin-Methylene blue staining of harvested esMCs from cytospins. (**C**) Morphology of esMCs by transmission electron microscopy. (**D**) Forward-side scatter plot of esMCs from hES cell line HUES-2. (**E**) CD14 surface expression on esMCs from hES cell line HUES-2. Histograms show antibody staining (in black) relative to isotype-matched control (in gray). (**F**) Accumulated esMCs yield and production kinetics from separate experiments from hES cell line HUES-2. Mean number of EBs used per experiment was 30 ± 13 EBs (range, 19–55, n = 9). (**G**) Phase contrast image of hES monocyte-derived macrophages (esMDMs) differentiated in M-CSF for 7 days and the esMDM expression of CD68 and CD163. Histograms show antibody staining (in black) relative to isotype-matched control (in gray). All scale bar units are in micrometers.

For embryoid body (EB) formation (Fig. 1A, Step 1), hES cells were removed from feeder cells either mechanically by microdissecting hES colonies into 0.2-mm–diameter patches, or enzymatically by dissociating colonies using trypsin for 2 minutes. Patches were transferred into six-well ultra-low adherence plates (Corning) in hES culture medium and cultured for 3 days at 37°C. For differentiation (Fig. 1A, Step 2), 50 to 100 EBs were transferred into tissue culture T25 flasks containing 10 mL culture medium supplemented with M-CSF and IL-3. Alternatively, 20 to 50 EBs were transferred into one well of a six-well plate in 4 mL medium. Medium was replaced every 5 to 7 days. Culture medium consisted of Advanced Dulbecco's modified Eagle's medium (Invitrogen GIBCO) and 10% fetal calf serum (Invitrogen GIBCO or Hyclone), supplemented with 50 ng/mL M-CSF (R&D), 25 ng/mL IL-3 (R&D), 2 mM l-glutamine (Invitrogen GIBCO), 100 U/mL penicillin and 100 μg/mL streptomycin (Invitrogen GIBCO), and 0.055 mM β-mercaptoethanol. For monocyte harvest and continuous production (Fig. 1A, Step 3), the supernatant was removed and replaced with fresh culture medium. Viability of esMCs was consistently greater than 90% by Trypan blue dye exclusion.

### Isolation of blood monocytes

Adult human blood was obtained from anonymous donors through the UK National Blood Bank Service, and tested negative for HIV-1, hepatitis B/C, and syphilis. Peripheral blood mononuclear cells were isolated by Ficoll-Hypaque (Pharmacia-Amersham) density-gradient centrifugation from heparinized buffy coats. Monocytes were isolated by CD14-positive selection using anti-CD14 magnetic beads (Miltenyi Biotec) by following manufacturer's instructions.

### Histochemistry of monocytes

Cytospins (400 rpm, 5 minutes) of hES monocytes were dried overnight. Slides were then sequentially fixed in methanol (30 seconds), stained with methylene blue (30 seconds) and stained with eosin (2 minutes). Slides were washed with water and images taken using a Nikon Coolscope.

### Differentiation of monocytes into macrophages

Human ES cell-derived monocytes or blood monocytes were differentiated into macrophages at a density of 1.5 × 10^5^ cells/cm^2^. Culture medium consisted of RPMI (Invitrogen GIBCO) and 10% fetal calf serum (Hyclone), supplemented with 100 ng/mL M-CSF (R&D), 2 mM l-glutamine (Invitrogen GIBCO), 100 U/mL penicillin and 100 μg/mL streptomycin (Invitrogen GIBCO). Medium was typically changed every 3 to 4 days by removing half and replacing culture media with twice the final concentration of M-CSF.

### Microscopy

Phase-contrast images were obtained using either an inverted-phase Axiovert 25 (Zeiss) or an Elipse TS1000 (Nikon) microscope. Images were processed using Corel Photo Paint and Corel Draw software.

For TEM and SEM, 3 × 10^5^ or 5 × 10^5^ cells were grown on 13 mm^2^ coverslips in a 24-well tissue culture plates and fixed in 2.5% glutaraldehyde, 2% paraformaldehyde, and 0.1% picric acid made up in 100 mM phosphate (pH 7.0). For TEM, coverslips were removed and remaining adherent cells postfixed in 1% osmium in 100 mM phosphate (pH 7.0) solution for 1.5 hours at 4°C, stained en bloc with 2% aqueous uranyl acetate for 2 hours at 4°C in the dark, dehydrated with ethanol and released from the plastic using propylene oxide. The released cells were pelleted and embedded in epoxy resin. Ultrathin (∼70 nm thick) sections were stained with uranyl acetate and lead citrate and examined in a FEI Tecnai 12 electron microscope.

For SEM, fixed cells on coverslips were rinsed several times with distilled water, dehydrated through an ethanol series and critical-point dried. After sputter-coating with gold, the samples were examined in a JEOL JSM 5510 microscope. All EM supplies were obtained from Agar Scientific.

### Flow cytometry

Cells were washed and stained in FACS buffer consisting of phosphate-buffered saline (PBS), human IgG (10 μg/mL, Sigma), 1% fetal calf serum (Hyclone), and 0.01% sodium azide. Macrophages were detached using cold PBS containing 5 mM ethylenediamine tetraacetic acid, washed and stained for surface markers on ice for 30 minutes. For intracellular staining, cells were fixed in 2% formaldehyde, permeabilized with 0.2% saponin and stained for 45 to 60 minutes. Cells were washed three times before acquisition, and all antibodies were compared with an isotype-matched control. Antibodies used included CD14-phycoerythrin (PE) (clone MEM-18), CD4-fluorescein isothiocyanate (FITC) (clone MEM-241), CD33-FITC (clone MD33.6) from Immunotools, CD68-FITC (clone KiM7), TLR2-FITC (clone TLR2.3), TLR4-FITC (clone HTA125) from AbD Serotec, and CD86-FITC (clone 37301.111) from R&D. Data were analyzed using FlowJo software and presented as histograms with antibody staining in black relative to isotype-matched control in gray. The percentage of cells expressing markers was determined by subtracting the isotype background from the antibody staining percentage. Protein expression levels were determined by dividing the geometrical mean fluorescence intensity (MFI) of the antibody staining with the MFI of the isotype control.

### Transcriptome analysis

The transcriptional profile was evaluated in three independent cell preparations using the GeneChip Human Gene 1.0 ST Array (Affymetrix) containing a total of ∼29,000 transcripts. For the esMC and hES monocyte–derived macrophages (esMDM) samples, RNA was purified from three different experiments using the HUES-2 hES cell line. For the blood–derived monocytes (bMC) samples, RNA was purified from three different donors. RNA was extracted with the RNeasy minikit (Qiagen) and RNA quality controlled by running samples on an Agilent Bioanalyser 2100. RNA integrity numbers were in the range of 8.8 to 10. RNA labeling, hybridization, and array scanning were conducted at the Sir Henry Wellcome Functional Genomic Facility at the University of Glasgow and signal values were generated using the Robust Multichip Average method [17]. Data sets from 11 human tissue samples were obtained from the Affymetrix Web site. Correlation analysis was done using the Affymetrix Expression Console software. Statistical analysis and hierarchical clustering were done using the TM4 software [18].

### Acetylated low-density lipoprotein endocytosis

Macrophages were washed with cold PBS and incubated for 1 hour at 37°C with OPTIMEM medium alone or in the presence of 100 μg/mL scavenger receptor ligand poly inosinic acid or control poly cytidylic acid. Medium was aspirated, and 2.85 μg/mL acetylated low-density lipoprotein (acLDL) added for 1 hour at 37°C. Cells were washed twice, analyzed by immunofluorescence microscopy and subsequently detached, fixed in 2% formaldehyde and analyzed by flow cytometry. Ratio of endocytosis uptake was calculated by dividing the MFI with the background signal from cells cultured without acLDL.

### Zymosan phagocytosis

Zymosan yeast particles were opsonized with 25% non-heat–inactivated human pooled serum (UK National Blood Bank) for 30 minutes at 37°C. Zymosan was added at a ratio of 50 particles per cell, to macrophages cultured on 13 mm^2^ glass coverslips, and incubated for 45 minutes at 37°C. Cells were washed twice in PBS before TEM. Phagocytosis was quantified by manual counting the number of particles observed per cell section.

### Cytokine and chemokine secretion

Human ES macrophages were incubated with 1 μg/mL lipopolysaccharide (LPS). Supernatants from cultures were harvested after 2, 8, and 10 hours and frozen at –20°C until assayed. Human tumor necrosis factor–α (5.9–95,949 pg/mL), IL-1rα (1.6–25,493 pg/mL), IL-1β (2.5–40,738 pg/mL), IL-6 (1.7–28,299 pg/mL), IL-10 (1.5–23939 pg/mL), IL-12(p70) (2.7–44,499 pg/mL), interferon-inducible protein 10 (3.3–53,434 pg/mL), monocyte chemotactic protein-1 (2.0–32,695 pg/mL), and eotaxin (0.2–2763 pg/mL), were measured using a tailored multiplex cytokine assay system (Bio-Rad Laboratories Hercules, CA, USA) [19]. Data analysis was carried out using Bio-Plex Manager software (Bio-Rad Laboratories) with a 5-parametric-curve fitting.

### Macrophage activation with IFN-γ and IL-4

Macrophages were activated with either 20 U/mL or 200 U/mL IFN-γ (R&D), or 20 ng/mL IL-4 (R&D) for 72 hours. The effect of activation was evaluated by quantifying changes in surface receptor expression of major histocompatibility complex (MHC) class II and mannose receptor by flow cytometry.

## Results

### A three-step method for the differentiation of hES cells into monocytes

Following detachment of hES cells from feeder cells, hES cells aggregate in suspension cultures to form structures known as embryoid bodies (EBs), which are composed of cells from all three germ layers [20]. To generate EBs, hES cells maintained on mouse embryo fibroblasts (MEF) were detached from MEFs and cultured in suspension for 3 days in hES medium (Fig. 1A, Step 1). For the directed differentiation into monocytes (Fig. 1A, Step 2), EBs were plated onto plastic and cultured in serum-containing medium supplemented with human recombinant M-CSF and IL-3. Monocyte-like cells were first observed migrating from the EBs and interacting with the adjacent EB-derived stroma (Fig. 1A, Step 3). These cells, which we refer to as hES-monocytes (esMCs), were harvested from the supernatant of cultures, have a ruffled membrane, high cytoplasm-to-nucleus ratio and typically an indented nucleus (Fig. 1B and 1C). esMCs consisted of a single, homogenous cell population based on morphology (Fig. 1B), the cell scatter properties (Fig. 1D and Suppl. Fig. 1) and CD14-expression profile (Fig. 1E and Suppl. Fig. 1). Furthermore, esMCs were phenotypically similar to blood monocytes isolated by positive selection for CD14, and expressed various leukocyte and monocyte receptors including CD45, CD44, CD11a, CD11b, CD11c, and CD54 (Suppl. Fig. 1). esMC did not express markers of dendritic cells such as CD83, integrin αE (CD103) or of blood stem cells such as CD34 (Suppl. Fig. 1). esMC purity, as determined by CD14-expression, was 90.9% ± 2.5% (mean ± SEM, n = 16) from cells derived from both hES cell line KCL002 (97.5% ± 0.8%, n = 2) and HUES-2 (90.0% ± 2.8%, n = 14). esMCs were produced after a lag period (mean 18.7 ± 7.7 days, n = 19), followed by a rapid production phase and a subsequent period of continuous lower level production that lasted for several months (Fig. 1F and Suppl. Fig. 1). Average esMC yield was 8.7 × 10^6^ ± 4.0 × 10^6^ (n = 9) cells with an average of 3.4 × 10^5^ ± 2.0 × 10^5^ esMCs produced per EB. In vivo, monocytes differentiate into macrophages when leaving the circulation and migrating into specific tissues, a process that is mimicked in vitro by allowing blood monocytes to adhere and differentiate on tissue-culture treated plastic. To test if esMCs could differentiate into esMDMs, we cultured esMCs on tissue-culture treated plastic for 6 days with M-CSF and evaluated cellular morphology and the expression of CD68, a marker expressed by the majority of human tissue macrophages [21,22] and CD163, a scavenger receptor expressed by M-CSF polarized macrophages [23]. We found that esMCs differentiated into a homogenous adherent and elongated macrophage population with a uniform expression of both CD68 and CD163 (Fig. 1G), similar to blood monocyte–derived macrophages (bMDM) cultured in M-CSF ([24] and data not shown). esMDM were phenotypically similar to bMDM and had downregulated CD14, expressed M-CSF receptor, Fc-γ receptor (FcγR) Type I (CD64), FcγR Type II (CD32), and CCR5 (Suppl. Fig. 1).

### Transcriptome analysis of esMCs and esMDMs reveal a distinct myeloid gene signature

To better characterize the esMCs and esMDMs, we studied the global expression of mRNA transcripts in these two cell types and compared the expression pattern relative to that of primary adult bMCs and several major human tissue samples including spleen, thyroid, brain, breast, heart, kidney, liver, pancreas, prostate, skeletal muscle, and testis.

**Figure 2:**
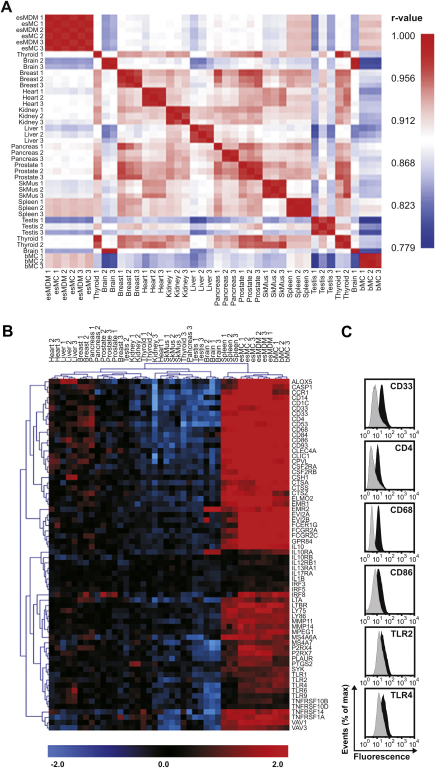
Transcriptome analysis of human embryonic stem (hES) monocytes (esMCs) and hES monocyte–derived macrophages (esMDM) reveal a distinct myeloid gene signature. Transcriptome analysis of esMCs using the GeneChip Human Gene 1.0 ST Array (Affymetrix) containing a total of ∼29,000 transcripts. (**A**) Pearson correlation analysis of RNA expression levels corresponding to esMCs, hES monocyte-derived macrophages (esMDMs), blood–derived monocytes (bMCs), and 11 other major tissues samples. Pearson correlation levels >0.9 are visualized in red, and <0.9 in blue. (**B**) Hierarchical clustering of 42 selected genes expressed in the esMC, esMDM, bMC, and spleen tissue samples. The 42 selected genes were obtained by performing a Euclidian distance complete hierarchical clustering with an adjusted Bonferroni corrected p value <0.05. (**C**) Confirmation of expression data of six selected proteins by flow cytometry.

We found that expression patterns in esMCs, esMDMs, and bMCs correlated significantly (r-value > 0.9) (Fig. 2A). We also found that while several of the tissue samples correlated significantly, only the spleen tissue samples correlated significantly (r-value > 0.9) with the esMCs, esMDMs, and bMCs (Fig. 2A). Since human spleen is highly enriched in mononuclear phagocytes, such as macrophages (about 16%) [25], the data suggest that the esMCs and esMDMs are similar to primary adult mononuclear phagocytes.

We next evaluated genes differentially expressed in esMCs, esMDMs, bMCs, and the spleen tissue sample compared with the remaining tissue samples. We found that 9.3% of the total genes (2699 genes) were differentially expressed and that 5.1% of the genes (1462 genes) represented a distinct myeloid signature. A selection of myeloid-related genes is shown in Figure 2B and we have confirmed the expression of several of these genes at the protein level by flow cytometry (Fig. 2C and data not shown). We found that esMCs and esMDMs expressed well-defined markers of the mononuclear phagocyte system including transcription factors PU-1 (SPI1) and C/EBPα [6,26], membrane receptors EMR1 and EMR2 [2,27–29], MPEG1 [30], CD1c [31], CD4 [32], CD18 (integrin β2), CD32 (FcγRII), CD33 (SIGLEC-3) [33,34], CD68 [21,22], and enzymes including the cathepsins [35], and serine carboxypeptidase (CPVL) [36,37]. In addition, esMCs and esMDMs expressed several genes involved in the recognition of the endotoxin LPS such as CD14, TLR2, TLR4, and LY86 (MD-2) [38,39].

Together, the data suggest that esMCs and esMDMs share a distinct myeloid gene signature with primary adult bMCs and spleen cells.

### esMCs differentiate into functional macrophages

Mononuclear phagocytes are phenotypically and functionally heterogeneous as a result of cellular differentiation, tissue distribution and responsiveness to various stimuli [3]. However, adult primary macrophages share functions including the ability to endocytose, phagocytose, secrete cytokines, and responsiveness to different activation signals. In order to test whether esMCs could differentiate into functional macrophages, we differentiated esMCs into esMDM and carried out various functional assays.

**Figure 3:**
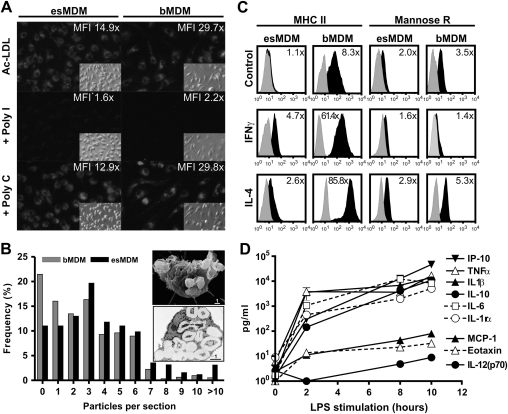
Human embryonic stem (hES) monocytes (esMCs) differentiate into functional macrophages. (**A**) hES monocyte–derived macrophages (esMDM) endocytose acetylated low-density lipoprotein (LDL). esMDMs and blood monocyte–derived macrophages (bMDM) were pretreated in medium alone, with the scavenger receptor inhibitor poly inosinic acid, or control poly cytidylic acid and cells incubated with acetylated LDL. Images show immunofluorescence with the corresponding phase-contrast image inserted. Inserted values show ratio of endocytosis uptake calculated by dividing the mean fluorescence intensity (MFI) with the background signal from cells cultured without acetylated low-density lipoprotein (acLDL). (**B**) esMDMs phagocytose opsonized yeast particles. esMDMs and bMDMs were incubated with zymosan particles opsonized with human serum at a 1:50 cell-to-particle ratio. Frequency distribution histogram show number of particles per TEM section comparing esMDMs (in black) with bMDMs (in gray). Inserted images show binding and internalization in esMDMs by SEM (top) and TEM (bottom). Scale bar units are in μm. Results are representative of two separate experiments. (**C**) esMDMs upregulate major histocompatibility complex (MHC) class II and mannose receptors after alternative activation with interleukin (IL)-4. esMDMs and bMDMs were cultured in media alone or activated with interferon (IFN)-γ (20 U/mL) or IL-4 (20 ng/mL) for 72 hours and assessed for MHC class II and mannose receptor surface expression by flow cytometry. Histograms show antibody staining (in black) relative to isotype-matched control (in gray). Inserted values show the ratio of surface receptor expression relative to isotype-matched control. (**D**) esMDMs produce chemokines and cytokines in response to lipopolysaccharide (LPS). esMDMs were cultured with LPS (1 μg/mL) and supernatants harvested after 2, 8, and 10 hours of stimulation and assessed for cytokine and chemokine production. Culture media contained less than the lower detection limit for all proteins tested. Results are mean values from experimental triplicates.

To test whether esMDMs could endocytose, we cocultured cells with fluorescently labeled acLDL. We found that esMDMs could endocytose acLDL nearly as efficiently as bMDM cultured under identical conditions (esMDM 14.9-fold vs bMDM 29.7-fold; Fig. 3A). We confirmed that endocytosis was mediated by scavenger receptors, because uptake could be inhibited specifically by a scavenger receptor inhibitor (Fig. 3A).

To test whether esMDMs could phagocytose, we cultured cells with yeast particles opsonized with human serum. We found that esMDMs were highly phagocytic with 89% of cells ingesting one particle or more, compared to 79% of bMDMs (Fig. 3B).

To test whether esMDMs could recognize and respond to a bacterial stimulus, we stimulated esMDMs with the endotoxin LPS, a bacterial ligand for the receptors CD14, TLR2, TLR4 and LY86 (MD-2) [38,39], which were found to be expressed in the esMC and esMDM (Fig. 2 and data not shown). We found that esMDMs produced high levels of tumor necrosis factor–α (16.8 ± 7.4 ng/mL), IL-10 (11.1 ± 5.6 ng/mL), IL-6 (>8.3 ng/mL), IL-1rα (5.1 ± 0.8 ng/mL), monocyte chemotactic protein-1 (>14.1 ng/mL) and interferon-inducible protein 10 (>46.5 ng/mL) after 10 hours of LPS stimulation (Fig. 3D). esMDM did not produce IL-1β, eotaxin or IL-12(p70).

To test whether esMDMs could be activated along both the classical and alternative pathways [40], we cultured cells in the presence of the T cell cytokines IFN-γ or IL-4 and evaluated the surface expression levels of MHC class II and mannose receptor. We found that esMDMs, like bMDM, upregulated surface expression of MHC class II after both IFN-γ and IL-4 activation, while only IL-4 activation increased mannose receptor expression (Fig. 3D).

In summary, we have developed an efficient method to produce hES-derived monocytes that differentiate into fully functional macrophages that can be used as a tool to study monocyte and macrophage development and biology.

## Discussion

The human mononuclear phagocyte system remains poorly understood due to limitations in current technologies. Methods for obtaining human primary myeloid progenitors, monocytes and macrophages for in vitro studies are inconsistent and differ in cell yield, purity, and activation status of cells [7]. Furthermore, primary monocytes and macrophages are inherently difficult to transfect. This limits the ability to genetically manipulate monocytes and macrophages to investigate their development and biology. Pluripotent ES cells offer an attractive alternative to overcome these problems and mES-derived mononuclear phagocytes have been successfully produced and studied in vitro [8–12]. Current methods using similar approaches with hES cells are complicated by the requirements for cocultures with other cell lines, the use of complex cytokine cocktails and additional purification steps. For example, the coculture of undifferentiated hES cells with mouse bone marrow cells or yolk sac endothelial cells produces hematopoietic progenitors that can be isolated by selecting for various markers and further differentiated into defined hematopoietic lineages [41]. Similarly, directed differentiation of hES using multiple cytokines also produces hematopoietic progenitors that can be differentiated into most hematopoietic lineages including erythroid, lymphoid, and myeloid cells [13,14,42–45].

In this study, we report a much simpler and highly efficient three-step method for the differentiation of hES cells into a homogenous population of esMCs. We found that esMCs were produced after approximately 18 days of directed differentiation in medium containing just two exogenous growth factors, and esMC continued to be produced for several months when growth factors were replaced.

We have validated this method using different hES cell lines derived from independent laboratories and confirmed esMC purity by morphology, scatter properties, and receptor expression profiles. In agreement with the adult mononuclear phagocyte system, the esMCs differentiated into functional macrophages and shared a distinct myeloid gene signature with both primary adult bMCs and spleen cells. Together, the data suggest that the method provides a good model system to investigate mononuclear phagocytes in vitro and could also be useful in identifying novel markers of the human mononuclear phagocyte system [2].

esMDMs were found to be functionally comparable to in vitro M-CSF–polarized adult bMDMs and shared many properties with these cells including morphology [24], expression of CD163 [23], high capacity to phagocytose and endocytose [46], and secretion of IL-10 but not IL-12 [23,47]. Interestingly, recent work has shown that M-CSF–polarized bMDMs share these functional characteristics with human peritoneal macrophages [46]. Because esMDMs maintain a stable and functional phenotype with time and the method can be scaled up to produce large number of cells, it will be particularly useful for investigating macrophage-specific functions in various diseases including atherosclerosis, sepsis, cancer, tuberculosis, and HIV-1.

In summary, we have developed an efficient method to produce esMCs that differentiate into functional macrophages which should prove useful in answering experimental questions regarding human monocyte and macrophage development and biology especially when combined with specific genetic modification of the hES cells.
