# Supplementary figures and images for: Homogeneous monocytes and macrophages from human embryonic stem cells following coculture-free differentiation in M-CSF and IL-3
Source: Exp Hematol. 2008 Sep;36(9-2):1167–75. doi: 10.1016/j.exphem.2008.04.009 (PMC2635571; doi:10.1016/j.exphem.2008.04.009)

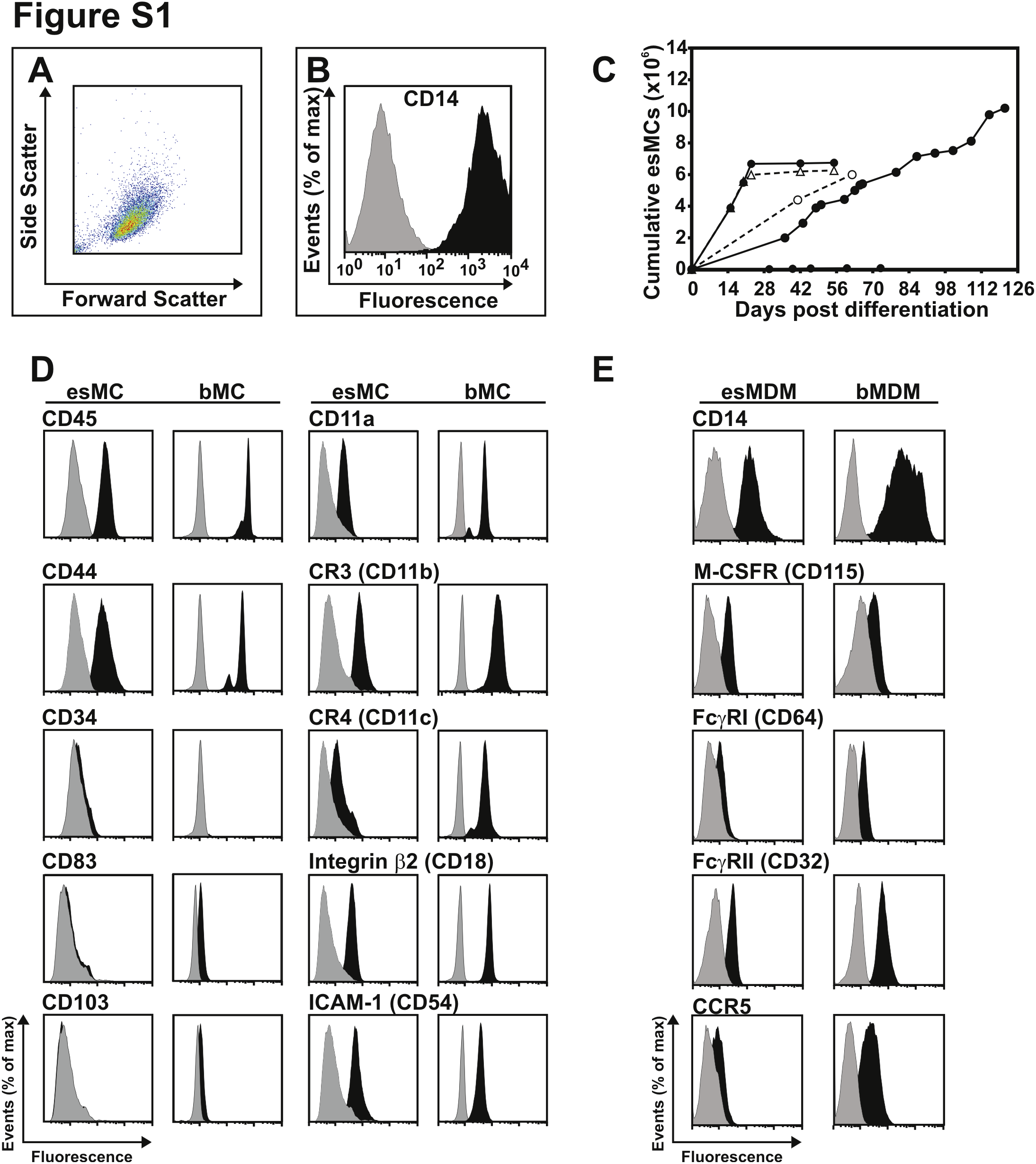

Supplement: Supplementary file 1 [file mmc1.tif]
